# Reactive Oxygen Species: Beyond Their Reactive Behavior

**DOI:** 10.1007/s11064-020-03208-7

**Published:** 2021-01-13

**Authors:** Arnaud Tauffenberger, Pierre J. Magistretti

**Affiliations:** King Abdullah University of Science and Technology, Thuwal, 23955 Kingdom of Saudi Arabia

**Keywords:** Reactive species, ROS, Hormesis, Homeostasis

## Abstract

Cellular homeostasis plays a critical role in how an organism will develop and age. Disruption of this fragile equilibrium is often associated with health degradation and ultimately, death. Reactive oxygen species (ROS) have been closely associated with health decline and neurological disorders, such as Alzheimer’s disease or Parkinson’s disease. ROS were first identified as by-products of the cellular activity, mainly mitochondrial respiration, and their high reactivity is linked to a disruption of macromolecules such as proteins, lipids and DNA. More recent research suggests more complex function of ROS, reaching far beyond the cellular dysfunction. ROS are active actors in most of the signaling cascades involved in cell development, proliferation and survival, constituting important second messengers. In the brain, their impact on neurons and astrocytes has been associated with synaptic plasticity and neuron survival. This review provides an overview of ROS function in cell signaling in the context of aging and degeneration in the brain and guarding the fragile balance between health and disease.

## Introduction

The cellular functions rely on a variety of extracellular signals and intracellular signaling that function in concert to maintain cellular homeostasis. Most, if not all, cellular processes require considerable energy. Mitochondria are known to fulfill this crucial role, producing the majority of the energy supporting cell growth and homeostasis. However, the dark side of energy production is the formation of reactive oxygen species (ROS) as a by-product by the mitochondria’s electron transport chain [1]. Until recently, ROS were essentially considered to be responsible for significant cellular damages [2], causing premature aging and neurodegenerative disorders. Since the 1950s and Harman’s *Free-radical theory of aging* [3], a compelling amount of research has investigated how ROS and reactive nitrogen species (RNS) influence disease progression. However, this theory is now being challenged on the basis of considerable evidence suggesting that ROS can act as second messengers. Furthermore, antioxidants that purportedly should antagonize the putative oxidative damage produced ROS have largely been ineffective in preventing disorders in which ROS are the considered as being the cause [4–7]. It is clear that ROS have complex influences on the cells, depending on their concentration. While their role in macromolecular damage and cell death upon loss of redox homeostasis is still a valid model, a mild increase of reactive species triggers various cellular signaling cascades that allow cell growth and survival [8–10]. Recently the concept of hormesis (which can also be dubbed “what does not kill you makes you stronger”) has been applied to ROS. Indeed, a contained production of these reactive species promotes stress resistance and longevity in model organisms such as *Caenorhabditis elegans* [11–13], *Drosophila melanogaster* [14, 15] and rodents [16].

## Nature of Reactive Species

ROS are, by definition, chemical molecules containing one oxygen atom that, through cellular and extracellular reactions become more reactive than oxygen itself. Reactive species are present in both radical, with and unpaired electron, and non-radical form. An example of ROS is the superoxide anion (O_2_^•−^) produced as a by-product of the mitochondrial respiration and NADPH oxidase activity. Other ROS include the hydroxyl radicals (OH^•^) and hydrogen peroxide (H_2_O_2_) a non-radical species. Another group is called RNS. Nitric oxide (NO^•^) is produced from l-arginine, by nitric oxide synthase (NOS) and acts a potent second messenger. NO promotes glycolytic metabolism by inhibiting mitochondrial respiration through cytochrome c oxidase and increased AMPK phosphorylation [17, 18]. In parallel, NO^•−^ interacts with superoxide (O_2_^•−^) to form peroxynitrite (ONOO^•−^) a highly reactive molecule capable of protein nitrosylation and target glutathione, a critical non-enzymatic antioxidant [17, 18].

## Sources of Reactive species

**Fig. 1:**
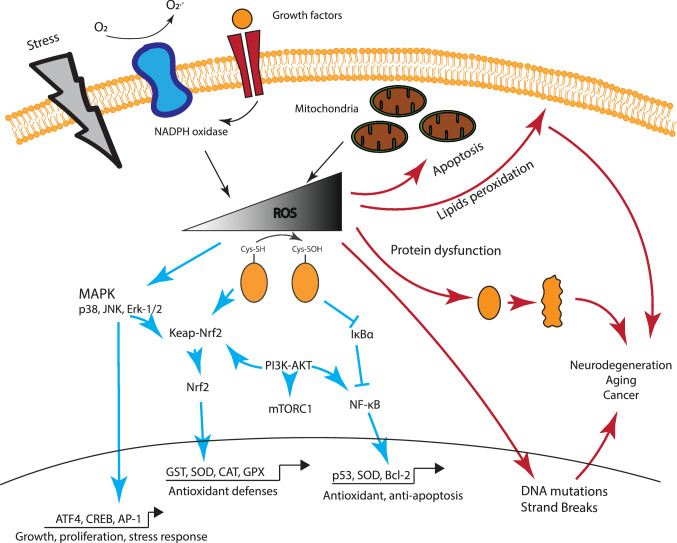
Schematic representation of the impact of ROS on cellular physiology. Low and Mild ROS level have a large impact on cell signaling, promoting activation of growth signals and kinases (Erk-1/2, PI3K, ATF4 and mTOR) and the transcription of pro-survival (Nrf2, PGC1α) factors. This interactive signaling culminates in the increased expression of antioxidant enzyme (SOD, CAT, GST), the effectors of the survival response. However, increased concentration of ROS disrupt cell signaling and activate pro-apoptotic signals in the mitochondria, as well as lipid peroxidation, protein oxidation and DNA damage. The accumulation of macromolecules and cell damage leads to a wide range of disorders and is associated with accelerate aging

Reactive species originate from two primary sources. ROS can either be released as by-products of oxidative metabolism, mainly through mitochondrial respiration or produced during cellular response to xenobiotics or cytokines released as part of a defense mechanism [19, 20] (Fig. 1). Energy production by the mitochondrial electron transport chain accounts for the majority of ROS in the cell. This leak of protons, originating from the oxidation of NADH and FADH_2_, at the complexes I (NADH dehydrogenase) and III (coenzyme Q and cytochrome c oxidoreductase) [21, 22] of the electron transport chain, produce a reduced oxygen ion known as superoxide (O_2_^•−^) [1].

The second primary source of ROS is the enzyme complex Nicotinamide adenine dinucleotide phosphate (NADPH) oxidase. Mammals possess seven NADPH oxidases (NOX1–5 and DUOX1–2) that produce ROS in the cytoplasm in response to a variety to stimuli. Initially identified in neutrophils, NADPH oxidase is a membrane-associated enzymatic complex involved in cellular signaling and disease through ROS production in the cytoplasm. Various ligands like TNFα, angiotensin II, PDGF and EGF [23–26] have been associated to NOX-mediated ROS production in response to cellular stimuli such as pathogen invasion, inflammation, growth factors and calcium signaling [27–29]. The complexes produce superoxide radicals and hydrogen peroxide, the latter being more stable and capable of diffusing through the cell membrane [30]. Another significant source of reactive species is the NOS. Present in different isoforms, these complexes are found as a constitutive form in neurons (nNOS or NOS1), as an inducible isoform in glial cells (iNOS or NOS2) and in the endothelial tissue (eNOS or NOS3). NOS produce nitric oxide (NO) that shapes the metabolic profile of the cell by inhibiting the mitochondrial respiration via inhibition of its complex IV (cytochrome c oxidase) and promoting glycolytic activity [31]. However, as mentioned previously, NO^•−^ also reacts with O_2_^•−^ to produce peroxynitrite (ONOO^•−^) a reactive specie involved in protein nitration, lipid peroxidation, and DNA damage. In addition of the mitochondria, NOS and NOX, other endogenous, and exogenous, sources have been linked to ROS production, such as the xanthine oxidase, cyclooxygenase, lipoxygenase and the cytochrome P450 [32–35], summarized in Table 1.

**Table 1:** Summary of the primary source of ROS

Source of ROS	Response stimuli	Pathway complexes	Main ROS
Mitochondria	Oxidative metabolism	Electron transport chain, NADH	O_2_^•−^
NADPH oxidase	Inflammation	NAPDH	O_2_^•−^
Xanthine oxidase	Purine catabolism	O_2_	O_2_^•−^
Nitric oxide synthase	Synaptic activity, inflammation, hypoxia	NADPH	NO, OONO
Peroxisomes	Lipid metabolism (β-oxidation)	NADH, NADPH, FADH_2_	H_2_O_2_, O_2_^•−^
Cytochrome P450	Clearance of various compounds (hormones, lipids, xenobiotics)	NADPH	O_2_^•−^
Lipoxygenases	Arachidonic Acid (PUFA) metabolism	O_2_	O_2_^•−^
Exogenous stress	Direct peroxidation, increased NOS, DNA damage	UV, environmental toxins, drugs	O_2_^•−^, ONOO^•−^, H_2_O_2_

## Antioxidant Mechanisms

Reactive species concentration need to be maintained at a low level to guarantee a proper cellular environment [36]; mechanisms that ensure antioxidant homeostasis are highly conserved across different species, from the simplest bacteria to humans. The endogenous antioxidant defense is composed of enzymatic and non-enzymatic factors. While the most reactive and toxic form of ROS is the superoxide radical (O_2_^•−^), its half-life is relatively short, and it does not diffuse far from the site of production. However, superoxide is quickly converted to hydrogen peroxide (H_2_O_2_), a more stable form of ROS that can diffuse through membranes. This conversion is mediated by superoxide dismutase (SOD). SODs come in three isoforms, located in different compartments. SOD1 (CuSOD) is mainly cytoplasmic, SOD2 (MnSOD) is located in the mitochondria and SOD3 (CuSOD) is an extracellular isoform. Loss of SOD is associated with an increased level of cellular damage such as lipid peroxidation and protein carbonylation. Mutations in SOD1 are also associated to familial cases of Amyotrophic Lateral Sclerosis (ALS), a devastating neurodegenerative disorder [37]. Although a high concentration of H_2_O_2_ in the cell can trigger cell death, a low concentration has been linked to several cellular processes related to cell development, growth and survival (see section “ROS Impact on Cell Signaling”). Accumulation of hydrogen peroxide is mainly limited by the activity of other types of enzymes, such as glutathione peroxidase (GPx) and catalases, active in the cytoplasm and the peroxisome respectively. The end-products of these enzymes are water and oxygen. The third ROS converted from H_2_O_2_, is the Hydroxyl radical (OH^•^), extremely active and oxidizing for lipids, proteins, and DNA [38–40].

Non-enzymatic antioxidants are molecules characterized by their capacity to inactivate reactive species quickly. The most common is glutathione (GSH), involved in both non-enzymatically reduction of ROS as well as being a cofactor in the glutathione peroxidase reduction of peroxides. The other primary non-enzymatic antioxidants include metal-binding proteins (albumin, ferritin, myoglobin, and transferrin) able to scavenge free radicals and metals [41–43], and coenzyme Q, a membrane-associated electron carrier involved in electron transfer capable of sustaining significant redox changes [44].

In parallel to the endogenous defense system, natural compounds like the flavonoids, polyphenols (flavonoids, phenolic acids), ascorbic acid (vitamin C) or α-tocopherol have antioxidant capacities that are important to ensure adequate protection against reactive species [45, 46].

## ROS Impact on Cell Signaling

At physiological concentrations, ROS have a broad spectrum of roles in signaling as second messengers, with a significant influence on physiological responses (Fig. 1). Several growth factors have been associated with an increase of ROS. Multiple external stimuli, including tumor necrosis factor- (TNF-), growth factors (PDGF, EGF) and cytokines, stimulate the formation of ROS. The main mechanism underlying ROS signaling is the oxidation of thiol (-SH) group on cysteine residues, an amino acid with a low p*K*a [47]. This reversible action regulates post-translational modification, alteration of protein activity, and relocation in a different cellular compartment.

ROS have been associated with an increased mitogen-activated protein kinases (MAPK) activity [48–50], either through activation of tyrosine kinases or oxidation–reduction of cysteine residues. MAPK are composed of three kinases playing a pivotal role relaying extracellular signals with important outcomes on cell growth, differentiation, development, cell cycle, survival, and cell death [51–53] The main MAPK pathways consist of extracellular signal-related kinases (ERK1/2), the c-Jun N-terminal kinases (JNK), the p38 kinase (p38). These serine/threonine kinases are activated by external stimuli (see above) or by environmental stress [54–57]. ROS influence other tyrosine phosphatases (PTP) and kinases (PK) that are sensitive to redox changes. These include PTEN, phosphatidylinositide 3-kinase (PI3K), AKT, and mTOR [58]. The PI3K–AKT axis plays an important role in cell growth, survival, and protein synthesis. Upon its activation by growth factors (EGF, PDGF) [8], PI3K promotes, and is influenced, by ROS production through NOX and mitochondrial activity, while ROS inactivate phosphatase PTEN [59, 60], PI3K’s primary inhibitor. Recently, the emergence of proteomic approaches has allowed identification of over 500 proteins sensitive to redox state, thereby demonstrating ROS capacity to deeply modulate cell activity [61].

ROS also impact the activity of important growth and metabolism-related transcription factors, sensitive to redox changes. The list includes, but is not limited to, Hypoxia Inducible Factor 1α (HIF-1α), NF-kB, Heat Shock Factor 1 (HSF1), p53 and nuclear factor erythroid 2-related factor 2 (Nrf2) [9, 10, 62]. Nrf2 and Kelch-like ECH-associated protein 1 (Keap1) are associated in the cytosol, promoting ubiquitination of Nrf2 and its degradation by the proteasome [63, 64]. However, ROS induce the oxidation of key reactive cysteine on Keap1, promoting the dissociation of Keap1-Nrf2, allowing translocation of the latter to the nucleus. There, Nrf2 engages with antioxidant response elements (ARE) and on the promoter region of antioxidant factors such as the Glutathione *S*-transferase (GST), leading to increased resistance to oxidative stress [65]. Overall, at low or moderate concentrations ROS play a role in signal transduction. They influence a variety of cellular pathways with a crucial impact on cell physiology, metabolism, and survival.

## Impact of Reactive Species on the Brain

The brain and, more specifically, neurons are susceptible to oxidative damage because of the high content of lipids and the heavy oxidative metabolism on which they rely [66]. Oxidative damages, through an accumulation of misfolded proteins and loss of antioxidant defenses, have been associated with the aging-mediated loss of functions [67] and neurodegenerative disorders such as Alzheimer’s disease (AD) and Parkinson’s disease (PD) [68].

In the brain, the different cell types are not equal regarding their resistance to oxidative stress. Thus glial cells, like astrocytes, are more resilient to oxidative insults, compared to neurons [18]. Similarly, neurons in different anatomical regions also display variability in their capacity to scavenge reactive species. Neurons in the amygdala, the hippocampus, and cerebellar granules cells appear to be the most sensitive [69, 70]. This sensitivity, compared to astrocytes for example, is also due to a low expression of antioxidant mechanisms [71]. Astrocytes synthetize most of the GSH content in the brain, express transcription factors such as Nrf2, at higher levels than neurons [72, 73] and clear ROS more efficiently [71]. Astrocytes release GSH that is either hydrolyzed to cysteine and used as a source for new GSH molecules in neurons via the γ-glutamate-cysteine ligase catalytic (Gclc) and modifier (Gclm) subunits and build antioxidant defense of their own [74–76]. There are several evidences supporting the role of astrocytes in organizing the antioxidant response through the release of cofactors or energy substrates to support neurons metabolism and synaptic activity [77–79]. Recently, some disputed work has shown that mild oxidative stress was able to stimulate astrocytes’ antioxidant defense through translocation of Nrf2, and promote neuronal survival [80, 81] but also that astrocytic ROS influence neuronal metabolism and improve survival [80, 82].

At synapses ROS are associated to long-term potentiation (LTP), to modulate plasticity and memory [83–85]. LTP is produced through high-frequency signals (HSF) resulting in activation of glutamate-activated *N*-methyl-d-aspartate (NMDA) receptors (NMDAR) permeable to calcium (Ca^2+^). Ca^2+^ entry triggers ROS production by the mitochondria [86] but also promotes nNOS activity [87, 88] through its binding to calmodulin leading to the formation of nitric oxide (NO^•−^). NO acts as a neurotransmitter, associated with synaptic plasticity and synaptic activity regulation through protein *S*-nitrosylation [89–95]. In astrocytes, induction of NOS2 is Ca^2+^ independent and can be triggered by external stimuli such as inflammation (LPS, TNFα, cytokines, Interferon-γ). Interestingly, NOS activity differs between neurons and astrocytes. NO synthetized in glial cells stimulates glycolytic function, while it does not induce a similar effect in neurons, despite similar capacity to inhibit mitochondrial respiration [96]. Besides direct synaptic regulation, ROS modulate the activity of a variety of protein kinases such as ERK, CAMKII, PKA, PKC involved LTP through transcriptional changes and increased number of glutamate (AMPA) transporters [97]. Manipulations aiming to reduce ROS production limit or abrogate LTP, strengthening the view that ROS have a signaling role in the brain [98–100].

## The Role of Reactive Species in the Periphery

Immune cells like macrophages and neutrophils release oxygen radicals upon phagocytic activity, potentially leading to tissue damage, yet these immune cells also are endowed with a high antioxidant capacity ROS are required for both innate and adaptive immune mechanisms [101, 102]. Reactive species are necessary for Lipopolysaccharide (LPS)-mediated activation of Toll-like receptor, leading to the production of pro-inflammatory cytokines [103]. Similarly, ROS can activate and maintain activation of lymphocytes (B and T) involved in the adaptive immune system, participating in its fine regulation. Furthermore, recent work has shown that the use of antioxidant can reverse these effects, leading to a deactivation of the immune system [104, 105].

In muscle cells, ROS play an essential role in contraction and adaptation to repetitive efforts [106]. As in other cell types, mitochondria are central for ROS formation; however in muscle cells, also NOX contributes significantly to reactive species formation both at rest and during exercise [107–110], resulting in particular in cell biogenesis through activation of peroxisome proliferator-activated receptor-g coactivator-1α (PGC-1α) [111]. However, excess levels of ROS induce a loss of contractile power that translates into muscle weakness and fatigue [112, 113]. The primary cellular mechanism involves the sustained activation of NF-kB and FoxO, leading to transcription of a degradation-related protein such as C/EBP homology protein (CHOP) [114–116]. Regular activity, however, can promote adaptation and increase muscle capacities (section “Beyond ROS Reactive Behavior”).

## Reactive Species in Aging and Disease

The principal harmful effect of ROS is observed during aging where a disequilibrium of the redox state is observed. With aging, neuronal metabolism is impaired, mainly through mitochondrial decay, resulting in decreased ATP and NAD^+^ production [117, 118]. This decrease, together with a failure in antioxidant defense mechanisms [119] leads to a rise in intracellular ROS-mediated dysfunction [120, 121]. Considerable evidence has demonstrated increased ROS levels in the nervous system of animal models of Alzheimer and Parkinson diseases or Amyotrophic Lateral Sclerosis [122–124]. Upon disruption of the redox homeostasis, ROS cause protein degradation [125–127], DNA damage [128, 129] and lipid peroxidation [130] (Fig. 1).

Accumulation of damage on macromolecules leads to cellular dysfunction, including in muscles and neurons. In tumoral cells, ROS promote stabilization of hypoxia-inducible factor 1α (HIF-1α), which in turn results in tumor survival by promoting angiogenesis and support of glycolytic metabolism [131–134]. Lipid peroxidation promotes inflammation and tissue damage in the heart and cardiovascular dysfunction [135].

Cancer cells are characterized by their “hyper-metabolism” linked to increased production of ROS [136], which is however neutralized by an equivalent increase in antioxidant defenses [137]. However, the role of oxidative stress-sensitive transcription factors such as Nrf2 is complex and depends greatly on the nature of tumors [138–140]. Altogether, it appears that cancer cells need to maintain a tight redox balance to maintain resistance to ROS. Among pro-tumorigenic factors, DNA mutations are associated to significant metabolic changes, that include reduced oxidative phosphorylation (OXPHOS) and increased glycolysis activity. Because ROS are mainly produced through OXPHOS, the diminution of ROS has been shown to promote tumorigenesis. Therefore, it appears that a minimal concentration of ROS is required for tumors to persist, and this concentration needs to be tightly regulated to prevent oxidative damage in cancer cells [141–144]. The high concentration of ROS has been at the center of attempts to develop therapeutic strategies against cancer, but the successes have been very limited or detrimental [145], suggesting that ROS are not a suitable target for therapies.

## Beyond ROS Reactive Behavior

Although ROS can have a deleterious effect on cell survival and in disease, their role in cellular physiology is more complex than initially subsumed. As mentioned above, ROS have a substantial impact on cellular signaling via regulation of over 500 redox-sensitive proteins, mainly kinases, and phosphatases that have a crucial effect on cell growth, differentiation and survival (see section “ROS Impact on Cell Signaling”). For example, it has been shown in multiple models that reduction of mitochondrial respiration can has a positive effect on longevity, in part due to a mild increase in ROS production. Caloric restriction is known to promote longevity and delay neurodegeneration: several observations suggest that ROS such as H_2_O_2_ could be linked to the positive outcome on longevity by activating anti-aging pathways such as the AMPK [146–148], while we and others have revealed a link between the protective effect of l-lactate against oxidative stress and ROS production [149, 150].

During moderate and repeated exercise, the production of ROS by muscle cells has a profound positive effect. Indeed it has been shown that a low concentration of H_2_O_2_ can increase muscle contractibility [151, 152]. A mild ROS increase can stimulate the expression of antioxidant enzymes, including GSH, but also SOD, CAT, and GPX. Endurance exercise, through a ROS-dependent mechanism, also reduces DNA damage [153] and increases insulin sensitivity [154]. This dose-dependent effect also translates into a long-term growth of the muscle fibers through activation of several signaling pathways such as AMPK, p38MAPK, and PGC-1α [155–157]. Interestingly, the use of exogenous antioxidant, through diet reduces the impact of ROS on muscle adaptation to exercise [158–160].

The latter observation is consistent with the role of preconditioning to ischemia as a protective strategy. Although, re-perfusion of tissue after hypoxia results in a dramatic ROS elevation and tissues damage, a small and short period of ischemia followed by reperfusion can produce protective effects, through ROS dependent mechanisms [161, 162].

## Conclusion

Reactive species are more complex than was initially thought. As of today, it appears that the equilibrium between pro-oxidant and antioxidant factors drives cellular physiology in multiple organs and organisms. While an excessive production of ROS has a dramatic negative effects on survival, a mild oxidative environment can produce a variety of positive outcomes crucial for biological organisms to survive and adapt. Therefore a better understanding of reactive species targets and effects, is necessary to target interventional strategies to improve major health-related issues.
